# Surgical treatment of detrusor underactivity: a short term proof of concept study

**DOI:** 10.1590/S1677-5538.IBJU.2016.0405

**Published:** 2017

**Authors:** Jerry G. Blaivas, James C. Forde, Jonathan L. Davila, Lucas Policastro, Michael Tyler, Joshua Aizen, Anand Badri, Rajveer S. Purohit, Jeffrey P. Weiss

**Affiliations:** 1 Department of Urology, Weill Medical College of Cornell University, New York, NY, USA;; 2 Department of Urology, SUNY Downstate Medical School, Brooklyn, NY, USA;; 3 Institute for Bladder and Prostate Research, New York, NY, USA

**Keywords:** Prostatic Hyperplasia, Urinary Bladder Neck Obstruction, Prostate

## Abstract

**Objectives:**

To compare the surgical outcomes of men with bladder outlet obstruction (BOO) due to benign prostatic obstruction (BPO) to those with detrusor underactivity (DU) or acontractile detrusor (DA).

**Materials and Methods:**

This retrospective, IRB approved study included men who underwent BPO surgery for refractory LUTS or urinary retention. Patients were grouped based on videourodynamic (VUDS) findings: 1) men with BOO, 2) men with DU and 3) men with DA. The primary outcome measure was the Patient Global Impression of Improvement (PGII). Secondary outcome measures included uroflow (Q_max_), post-void residual volume (PVR) and the need for clean intermittent catheterization (CIC).

**Results:**

One hundred and nineteen patients were evaluated: 1) 34 with BOO, 2) 62 with DU and 3) 23 with DA. Subjective success rate (PGII) was highest in the BOO group (97%) and those with DU (98%), while DA patients had a PGII success of 26%, (p<0.0001). After surgery, patients with BOO had the lowest PVR (68.5mL). Fifty-six patients (47%) performed CIC pre-operatively (47% of BOO, 32% of DU and 87% of DA patients). None of the patients in the BOO and DU groups required CIC post operatively compared to16/23 (69%) of patients in the DA group (p<0.0001).

**Conclusions:**

BPO surgery is a viable treatment option in men with presumed BOO and DU while DA is a poor prognostic sign in men who do not void spontaneously pre-operatively.

## INTRODUCTION

The goal of prostate surgery for bladder outlet obstruction (BOO) is to improve lower urinary tract symptoms (LUTS) in men by relieving benign prostatic obstruction (BPO). Its efficacy in men with proven BOO has been well documented (1, 2). Impaired detrusor contractility in the form of detrusor underactivity (DU) or detrusor acontractility (DA) can contribute to LUTS and confound the diagnosis of BPO. The diagnosis of DU can only be made by detrusor pressure-uroflow urodynamic studies (3). DU is defined by the International Continence Society (ICS) as, “a contraction of reduced strength and/or duration, resulting in prolonged bladder emptying, and/or failure to achieve complete bladder emptying within a normal time span” (4). This definition, though, is devoid of metrics; and does not specifically define “reduced strength,” detrusor contraction “duration”, and “a normal time span”.

It has been reported that as many as 48% of men being assessed for LUTS display evidence of DU (5). There is much lacking in our understanding of the underlying physiologic mechanisms of DU, which is likely to be multi-factorial in nature, with both myogenic and neurogenic etiologies. It is also generally recognized that detrusor contractility diminishes with aging (4, 6, 7), but in some cases DU co-exists with BPO and can be a result of long standing untreated obstruction. Levin et al., in experimental studies in humans and rabbits have demonstrated that obstruction can lead to the development of smooth muscle hypertrophy, which is associated with significant intracellular and extracellular abnormalities in the smooth muscle cell (8, 9). Specifically, they documented changes in contractile protein expression, abnormalities of calcium signaling, impaired cell communication and mitochondrial dysfunction. Those authors postulated that these findings were responsible for both detrusor instability and impaired detrusor contractility (8, 9).

At present, there are no clear methods of diagnosing BPO in men with DU unless detrusor pressure at maximum uroflow (pdetQ_max_) is > 40 cm H_2_0 and men with DU represent an underreported segment of the population of those with LUTS. In addition, there is much controversy in the surgical management of these cases as many urologists hesitate to consider prostate surgery in men with DU for fear that the results are suboptimal, unnecessarily subjecting them to the risk of the surgical procedure (10). In this study, we investigate this problem by comparing the outcomes of endoscopic prostate surgery in men with urodynamic evidence of BOO compared to those with either DU or DA.

## MATERIALS AND METHODS

This is a retrospective, IRB approved study of men who underwent endoscopic surgery for BPO at a single institution in the form of either a monopolar Transurethral Resection of the Prostate (TURP) or Photoselective Vaporization of the Prostate (PVP) using the potassium titanyl phosphate (KTP) laser. Indications for surgery were refractory LUTS thought to be due to BPO or refractory urinary retention. A database was searched for patients who underwent either of these procedures and also underwent preoperative videourodynamics (VUDS). The patients were divided into three groups based on videourodynamic findings, 1) men with BOO (defined by a Bladder Outlet Obstruction index (BOOI) > 40) (11), 2) men with DU and 3) men with acontractile detrusor (DA). DU was defined by a Bladder Contractility Index (BCI) < 100. Acontractile detrusor (DA) was defined as the absence of a detrusor contraction on VUDS despite filling to bladder capacity. In patients with equivocal findings (BOOI between 30-39), the urodynamicist made a clinical judgment based on detrusor contraction duration and magnitude and the radiographic appearance of the urethra during voiding.

Patients who were on CIC were advised to try to void before each catheterization and their ability to do so was recorded. All subjects had pre-operative uroflow (Q_max_), post-void residual volume (PVR) measurements, VUDS and cystoscopy. Post-operative Q_max_, PVR, need for clean intermittent catheterization (CIC), and Patient Global Impression of Improvement (PGII) (12) score were obtained at least 3 months and up to 12 months after BPO surgery. The Patient Global Impression of Improvement (PGII) is an instrument used to assess patient satisfaction following treatment for a given condition; the seven point scale rates outcomes from 1=very much better to 7= very much worse (12). The PGII has previously been used to validate the success of patients following BPO surgery (13). The AUA symptom score (AUASS) or the lower urinary tract symptom score (LUTSS) (14) were collected before and after surgery in men who were not catheter dependent preoperatively.

When multiple values of Q_max_ and PVR were available, the highest and lowest values were used, respectively. Subjective success was defined by a PGII score of 1-3 whereas failure (no change or worsening of symptoms) was scored 4-7. All available data parameters were compared using either unpaired non-parametric two-tailed t test or Kruskal-Wallis test. All analyses were performed using Prism Graphpad 5 (CA, USA).

## RESULTS

**Figure 1:**
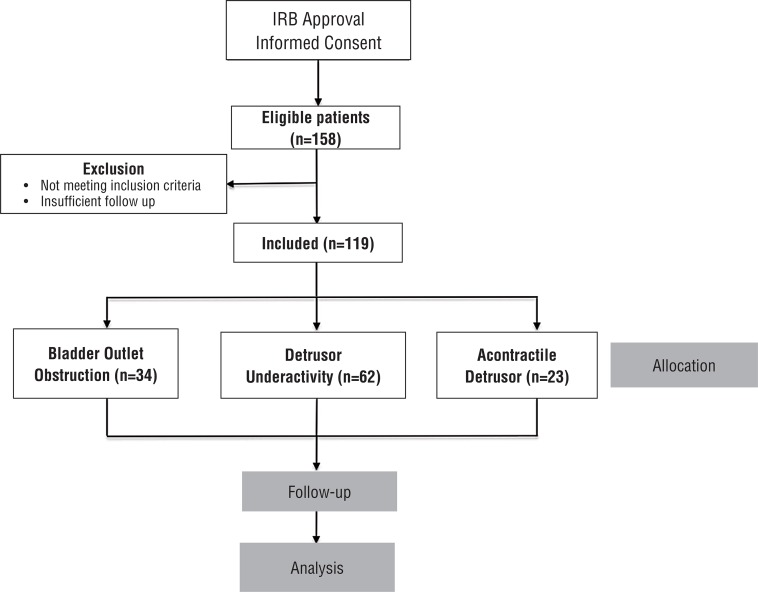
Patient selection.

In total, 157 men were identified who underwent surgery for BPO (Figure-1). Of these, 38 were excluded because of incomplete VUDS or missing PGII data. The remaining 119 were divided as follows; 1) 34 men with BOO, 2) 62 men with DU and 3) 23 men with DA. Follow up ranged from 3-12 months (mean 9 months). Table-1 shows the breakdown between the number of TURP and PVP procedures performed for the individual groups. From the total of 119 surgeries for BPO, TURP accounted for 57 procedures (48%) while 62 PVP procedures (52%) were performed.

**Table 1:** Type of BPO surgery performed in each group.

	BOO [n=34]	DU [n=62]	DA [n=23]
TURP (%)	11 (32%)	31 (50%)	15 (65%)
PVP (%)	23 (68%)	31 (50%)	8 (35%)

Table-2 shows that there was no difference in age, PVR or prostate volume between the three groups, but there was a higher pre-op Q_max_ in the BOO group (p<0.001). As expected both BCI and BOOI significantly varied between BOO and DU groups (p<0.0001).

**Table 2:** Preoperative data.

	BOO [n=34]	SD	DU [n=62]	SD	DA [n=23]	SD	P value
Age (in years)	67.9	12.4	68.4	11.9	71.6	11.8	0.47
BCI	124	22	54	26	--	--	<0.0001
BOOI	69	39	23	27	--	--	<0.0001
Pre-Op Qmax (mL/s)	7.8	5.2	4.4	3.7	--	--	<0.001
Pre-Op PVR (mL)	481	443	381	376	--	--	0.08
Bladder Capacity (mL)	553	302	619	392	1034	665	0.0001
Prostate volume (mL)	46	21	47	28	50	26	0.82

Table-3 shows the pre and postoperative results. The subjective success rate (PGII) was highest in the BOO (97%) and DU (98%) groups, while the DA patients had a PGII success rate of only 26% (p<0.0001). Comparison of AUASS and LUTSS scores before and after surgery revealed symptomatic improvements in the DU (p<0.025) and BOO (p<0.0006) groups. After BPO surgery, patients with BOO had a similar PVR to the DU group (68.5mL vs. 78.3mL, p=0.032). The change in Q_max_ after surgery was also similar between BOO and DU groups (11.1mL/s vs. 11.4mL/s, p=0.95). In both BOO and DU groups, improvements in Q_max_ and PVR after surgery were significant (p<0.0001).

**Table 3:** Comparison between Pre- and Post-Operative Outcomes.

Parameter	BOO [n=34]	DU [n=62]	DA [n=23]	P value
PGII success (%)	33/34 (97%)	61/62 (98%)	6/23 (26%)	<0.0001
Pre-op need for CIC (%)	16/34 (47%)	20/62 (32%)	20/23 (87%)	<0.0001
Post-op need for CIC (%)	0/34 (0%)**	0/62 (0%)**	16/23 (69%)*	<0.0001
%Δ in need for CIC	-100%	-100%	-17%	<0.0001
Pre-op Q_max_ (mL/s)	7.8	4.4	--	<0.001
Post-op Q_max_ (mL/s)	18.9**	15.9**	--	0.07
Δ Q_max_	11.1	11.4	--	0.95
Pre-op PVR (mL/s)	481.3	380.7	--	0.08
Post-op PVR (mL/s)	68.5**	78.3**	--	0.32
Δ PVR	-393.3	-295.5	--	0.09
Pre-op AUASS	21.6	15.3	16.0	0.22
Post-op AUASS	5.5**	9.5*	9.0	0.45
Pre-op LUTSS	26.4	21.7	27.0	0.54
Post-op LUTSS	12.5**	14.3*	16.4	0.72

* indicates pre vs. post p < 0.03

** indicates pre vs. post p < 0.003

Δ = The symbol means change.

Of the 119 patients, 56 (47%) were on CIC pre-operatively. This included 47% of BOO patients, 32% with DU and 87% of the patients in the DA group. Three men in the DA group were able to void spontaneously at home but unable to demonstrate this on VUDS. After surgery, a further 4 patients with DA no longer required CIC, leaving 16/23 (69%) patients in the DA group who were CIC-dependent post-operatively compared to none of the patients in the BOO and DU groups (p<0.0001). This improvement was significant in all groups (DA, p=0.01; BOO and DU, p<0.0001).

## DISCUSSION

Historically, impaired or absent detrusor contractions during urodynamics has been considered a poor prognostic sign for a successful outcome after BPO surgery in men with refractory LUTS (10, 15). The data presented herein suggests that outcomes do not differ between patients with and without DU undergoing BPO surgery. Specifically, there was no difference in outcomes after BPO surgery in men with DU and BOO versus BOO alone who can generate a detrusor contraction during VUDS. Preoperatively, men with BOO had higher Q_max_, but there was no difference between the degrees of improvement in parameters postoperatively.

Further analysis of data revealed that men with detrusor acontractility who never void spontaneously while on CIC have an overall poor prognosis. Urodynamic studies provide the physician with a snapshot of bladder function in a potentially intimidating environment, which may inhibit normal voiding function and may result in a spurious acontractile detrusor. We hypothesize that if a man is able to void between catheterizations while on CIC, he likely has retained at least some detrusor function and that BPO surgery will reduce outlet resistance and improve voiding mechanics. To wit, the data confirms a significant failure rate in patients on CIC who are never able to void spontaneously with only 26% of those patients having a successful outcome after BPO surgery.

Our literature search found a limited number of studies describing the outcomes of patients with DU after BPO surgery and, in fact, Thomas et al. in 2003 reported that they were unable to find a single relevant study when they reported their results on 22 patients with DU who had undergone TURP. Their study, with a mean follow-up of 11 years, found no clinical or urodynamic benefit from surgery (15). However, they did not report any patient reported outcomes like the PGII. Further, this study was highly selected in so far as only 22 at 284 patients with DU actually underwent TURP (15). A number of recent studies, however, showed much more encouraging results. Masumori et al. reported the long-term outcomes of a cohort of 92 men undergoing TURP (16). There were 34 patients who completed the 12-year follow-up including a subgroup of 12 patients with DU who reported a long-term benefit in terms of IPSS and QoL scores following surgery (16). Han et al. examined the effect of TURP in 25 men with weak bladder contractility compared to 46 men undergoing TURP with obstructed and/or normal bladder contractility and compared pre and post-operative IPSS, quality of life questionnaires and uroflowmetry (17). Groups were separated on a urodynamic basis using BOOI < 40 and BCI < 100 as criteria for inclusion into their DU group (17). They reported a 60% satisfaction rate among the 25 patients having poor bladder contractility with significant improvements in both voiding and storage parameters of IPSS and quality of life questionnaire (IPSS/QoL). Flow rates between groups did not differ, however, there was a significant reduction in post-operative PVR. Although patients with normal bladder contractility had significantly more improvement after TURP, outcomes were promising for those with evidence of impaired bladder contractility. Improvement in this group of patients was attributed to BOO, masked by the underlying DU, which was treated by resection, unrecognized by initial urodynamic study due to reduced detrusor pressure at the time of voiding. These findings were corroborated by van Venrooij et al. who reported that bladder outlet reduction in 34 patients with equivocally obstructed or unobstructed bladders produced a reduction in symptoms albeit to a lesser extent (70%) than 59 patients who were obstructed (18). They also document a significant 40% reduction in urethral resistance in the unobstructed group, which is a possible explanation for the improvement in those without obstruction (18).

In addition to these comparative studies, additional authors have also suggested that TURP is a viable option in patients with DU. Specifically, Ou et al. reported on their prospective cohort of 20 patients with BPH and urodynamically diagnosed detrusor “hypocontractility”, revealing significant improvements in IPSS/QoL, Q_max_, PVR and maximum P_det_ after TURP (19). Seki et al. retrospectively reviewed 190 patients with DU and assessed outcomes 12 months after TURP, concluding that only pre-operative level of storage symptoms in this group negatively impacted improvement post-operatively. However, peak urinary flow rates were positively influenced by baseline degree of bladder obstruction (20). Tanaka et al. examined the preoperative urodynamics of 92 men who underwent TURP and classified them as either BOO, DU and detrusor overactivity (DO) (21). There were 37 (40.2%) patients deemed to have weak/very weak contractility (18). They confirmed that a higher degree of bladder outlet obstruction predicts a better chance of improvement after TURP, but that presence of DU itself did not influence the likelihood of positive post-surgical outcome (21). In comparison to these published studies we included a significant number of patients with DU (62 patients or 52% of total patients involved) with a subjective success rate of 98%. The improvement in Q_max_ was comparable between both BOO and DU patients.

**Figure 2:**
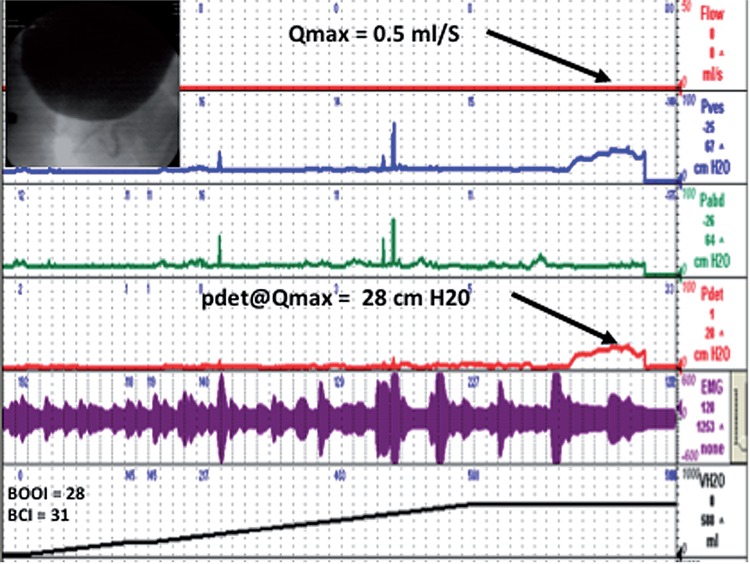
Sample VUDS of patient with DU.

**Figure 3:**
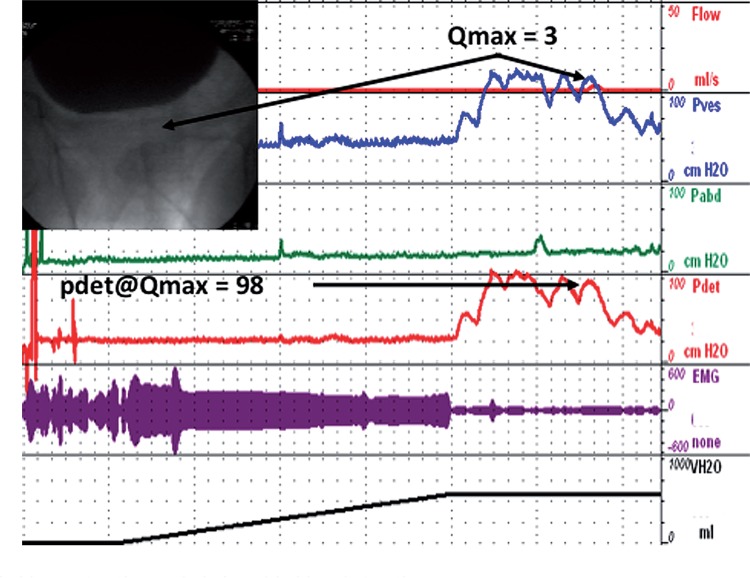
Sample VUDS of patient with BOO and normal detrusor function.

The utility of urodynamics in this setting has been called into question as it has been suggested that objective findings are generally inaccurate in predicting response to surgery (22, 23). We find urodynamics very useful in predicting the outcome of surgery, but our opinion is based largely on a qualitative assessment of the pressure flow curve and radiographic appearance of the urethra during voiding as depicted in the two videourodynamic tracings seen in Figures 2 and 3 comparing a patient with DU and BOO and a patient with BOO and normal detrusor function.

Example of patient with urodynamic evidence of detrusor underactivity. He has a low amplitude sustained contraction occurring for roughly 60 seconds. Pdet at Q_max_ is only 28 cm of water and the Q_max_ at this point is 0.5mL per second. According to BOO index calculation, he does not have urodynamic evidence of an obstruction.

Patient with a strong, high pressure bladder contraction and obvious bladder obstruction.

We believe that a sustained detrusor contraction and narrowed prostatic urethra portends a good outcome, but a much larger study is necessary to determine whether this is true. Some investigators have attempted to find features that can provide useful clinical information to guide those who question the efficacy of surgical intervention. Blatt et al. investigated ultrastructural features on detrusor biopsy in patients with detrusor failure after TURP and found that a combination of muscle cell size, shape, collagenosis and abnormal fascicles predicted postoperative voiding failure (24).

Although urodynamics has its limitations, it does provide useful information specifically in those who are found to have no detrusor function who never void spontaneously. Our data suggests that these patients are likely not going to benefit from surgery and should thus be considered for more conservative management (i.e. continuous or intermittent catheterization). However, the observation that occasional patients with DA do come off CIC keeps the surgical option open for this poor-prognosis group.

There are a number of limitations to this study. Because of its retrospective nature, it was not possible to determine how many surgical candidates were not offered or refused surgery. Further, there were different group sizes and relatively small numbers, but that is due, in part, to the fact that a smaller number of patients with impaired detrusor contractility undergo BPO surgery. The study included two different types of surgery for BPO (both TURP and PVP). Our hypothesis was to evaluate the effect of BPO surgery as an entity in patients with DU or DA compared to outcomes in those with proven BOO. We acknowledge that variations may exist in terms of technique between each procedure type. However, both standard electrosurgical TURP and PVP are well-established surgical treatments for BPO with the latter recently shown to ‘exhibit efficacy and safety outcomes similar to TURP’ in the recently published GOLIATH study (25).

Notwithstanding that, our cohort of DU patients is one of the largest published series to date. Follow up was limited to 3-12 months after surgery because we considered this study a proof of concept design and recognize that larger numbers and longer follow up is necessary to prove long term efficacy. Another limitation is the inherent bias generated as groups were constructed based on a clinical suspicion that there was an underlying obstruction that was not documented by urodynamics.

Despite the unstructured follow up, we believe that results of the study prove an important point, a proof of concept – that most men with detrusor underactivity have an underlying prostatic obstruction and that surgery designed to relieve the obstruction is effective in the majority of patients. The durability of the outcome remains in question^15^, though there have been reports of long-term benefit (20).

## CONCLUSIONS

BPO surgery is a viable treatment option in men with presumed BOO and DU. However, acontractile detrusor is a poor prognostic sign in men who do not void spontaneously while on intermittent catheterization. Prospective studies with larger patient cohorts would be beneficial to help confirm the findings of this study.

## ABBREVIATIONS

LUTS: Lower Urinary Tract Symptoms
BOO: Bladder Outlet Obstruction
BPO: Benign Prostatic Obstruction
DU: Detrusor Underactivity
DA: Acontractile Detrusor
VUDS: Videourodynamics
Q_max_: Maximum flow rate
Pdet: Detrusor pressure
PVR: Post Void Residual volume
PGII: Patient Global Impression of Improvement (PGII)
CIC: Clean Intermittent Catheterization
ICS: International Continence Society
IRB: Institutional Review Board
TURP: Transurethral Resection of the Prostate
PVP: Photoselective Vaporization of the Prostate
KTP: Potasium Titanyl Phosphate
BOOI: Bladder Outlet Obstruction Index
BCI: Bladder Contractility Index
AUA: American Urological Association
IPSS: International Prostate Symptom Score
AUASS: AUA Symptom Score
LUTSS: Lower Urinary Tract Symptom Score
QoL: Quality of Life
